# Urban Resources Selection and Allocation for Emergency Shelters: In a Multi-Hazard Environment

**DOI:** 10.3390/ijerph15061261

**Published:** 2018-06-14

**Authors:** Wei Chen, Guofang Zhai, Chongqiang Ren, Yijun Shi, Jianxin Zhang

**Affiliations:** 1 School of Geographic and Oceanographic Sciences, Nanjing University, Nanjing 210046, China; chenw.nju@gmail.com (W.C.); zhangjxnju@hotmail.com (J.Z.); 2 School of Architecture and Urban Planning, Nanjing University, Nanjing 210093, China; yijun_shi@smail.nju.edu.cn; 3 College of Economics, Northwest Minzu University, Lanzhou 730030, China; rcq518@163.com

**Keywords:** multi-hazard environment, urban resource, emergency shelter

## Abstract

This study explores how emergency shelters can adapt to a multi-hazard environment by geographic information system (GIS) and takes Guangzhou as a case for analysis. The physical suitability of the overall urban resources was first assessed by aiming to select the suitable resources and safe locations for emergency shelters in the context of multiple disasters. Afterward, by analyzing the scale and spatial distribution of affected areas and populations under different types of disaster scenarios, the demand for different kinds of shelters were predicted. Lastly, taking into account the coverage of the affected people, shelters were allocated according to different conditions in the districts. This work will hopefully provide a reference for the construction of emergency shelters and help form emergency operations in order to mitigate the impact of hazards. The issues identified in the study need to be further studied in medium or small-scale cities.

## 1. Introduction

The development and construction of cities are always accompanied by efforts to prevent various disasters that could directly or indirectly bring heavy losses to human society [1,2,3]. To some extent, human cognition of disasters and adaptation of the environment have been greatly improved. Strengthening disaster preparedness, risk analysis, emergency response, and management have become practical solutions in disaster relief works [4,5,6,7,8]. With lessons learned from disasters, it is of great significance to make a systematic planning program for disaster prevention, which should be continuously optimized as an important public policy [9,10]. The crucial issue in this work constructs emergency shelters, which are concrete materializations and visualizations of disaster preparedness and risk calculations [11]. Practices in disaster prevention show that urban emergency shelters can mitigate the impact of major natural disasters as well as play a significant role in post-disaster reconstruction [12,13]. In terms of space form, emergency shelter is basically outdoor and indoor shelters. The former mainly consists of parks, playgrounds, and squares where assisted living facilities are not necessary. The latter includes public infrastructure and buildings that are structurally better constructed such as gymnasiums and libraries. These buildings can provide living conditions and support facilities for refugees or vulnerable populations who need special care in disasters. The intensity of a disaster determines how long people will stay in a shelter. Indoor ones are more inclined to give people long-term refuge and outdoor ones can do so if enough tents and other living facilities are supplied [14,15,16]. Functionally, outdoor shelters are mostly used for disasters such as earthquakes, floods, and fires, and situations that deem indoor stay unsafe at the beginning of a disaster or during the entire process. In contrast, indoor shelters are usually used to respond to disasters such as hurricanes and storm surges that make the outdoors risky [15,16].

The selection of urban spaces for emergency shelters is a key point in planning shelters because candidate shelters may be unreliable for their unsuitable location [17]. For example, an assessment for Southern Florida found that 48% of the existing shelters and 57% of candidate shelters are located in physically unsuitable areas. In addition, 15 of the existing shelters are located in unsuitable areas with no alternatives [18]. Major factors, according to current studies on site selection of shelters, are summarized as follows: First, potential risk areas and geological hazards need to be excluded to ensure site suitability and safety. The shelters should be kept at a safe distance from gas stations, chemical warehouses, large slope areas, landslide points, and flood-submerged areas, where secondary disasters often occur [14,17]. Second, evacuation time should be reasonable. Studies show that people tend to take refuge in a familiar environment that offers emotional security and a sense of belonging [19,20]. This means that residential areas and work places should be considered as priorities to ensure that the potential evacuees can evacuate to emergency shelters as soon as possible. In addition, any other areas with high-population concentrations should also be taken into account. Therefore, a systematic assessment of candidate evacuation routes is required to estimate evacuation time as well as figure out the coverage of affected people [18,21,22]. Third, accessibility to emergency shelters is another critical issue in site selection. Evacuation routes could be inundated by flood or covered by collapsed buildings in an earthquake, which will reduce the efficiency of evacuation and result in major casualties. The main purpose of assessing shelter accessibility is to find out whether people can reach the shelter from their locations quickly after a disaster, providing feasible advice for evacuation route optimization, shelter construction, pre-disaster drills, emergency measures, and relief operations [14,17,23]. Lastly, the basic needs of evacuees should not be ignored. People experience physical and psychological stress and need support during an emergency. It is worse for vulnerable populations with special needs such as the elderly, children, and physically disabled people. Shelters located within close proximity of hospitals, medical centers, and other available public facilities can provide medical services and other forms of support, thus helping reduce the number of casualties in the event of a disaster [19,24].

Even after candidate shelters are selected, it is unclear how many people will actually take refuge in them. Not all the people affected by disasters make the decision to evacuate or follow evacuation orders by authorities [20,25]. The complexity and uncertainty of individual behaviors shown during disasters can be caused by external and internal factors such as the frequency of evacuation drills, regional economic development level, past experiences, risk perception, and family issues [26,27,28]. Limited understanding of behaviors of the affected people might influence the government’s disaster relief works, which range from shelter construction to emergency evacuation or refugee estimation. Additionally, the evaluation of shelter needs could deviate from the actual situation if potential behaviors are neglected [29]. However, the losses in a disaster are associated with the time it strikes, implying that the number of people affected differs from day to night. This also means that emergency shelters should service high-population areas with maximum efficiency, regardless of when disasters occur. Population distribution can be figured out by land-use type. For example, The number of people in an industrial park in the day is higher than at night [30,31]. The number of potential evacuees is estimated mainly by vulnerability analysis in a simulated disaster environment, without consideration for behavioral factors. [32]. During an earthquake, serious damage is often at the epicenter or in other hazardous areas with high-population and high-building density. Additionally, these areas are prone to have a great demand for emergency shelters as the magnitude of the earthquake increases [33]. In the case of tsunamis, early warnings and evacuation activities are the most important in limiting casualties. The number of affected people can be estimated by the population in submerged areas. Unlike earthquakes, the demand for tsunami shelters might come from small districts [34].

The allocation of emergency shelters aims to strike a balance between demand and capacity, which means candidate shelters need to provide refuge for disaster victims at a maximum coverage [30]. In other words, there is a price for the use of urban resources. The management department is also concerned about how to maximize the social benefits of emergency shelters so that they can be used for other purposes or be in perpetual use [35]. At present, methods proposed by mathematical models and the GIS platform focus on determining and optimizing the allocation of emergency shelters to respond to a variety of disasters such as earthquakes [36,37,38], floods [16,39,40] and hurricanes [15,41,42]. Serious disasters may damage the links between evacuation routes and shelters, making it difficult for evacuees to reach the shelter in time or fail to act in accordance with the original plan. Furthermore, it can result in disorderly evacuation and overload of other nearby shelters [43]. In addition, reliable shelters can reduce casualties to some extent and evacuees do not have to face the risk of insufficient food, medicine, and other support items. Emergency operations planning (EOP) needs to be considered to avoid post-disaster problems if a shelter is destroyed and unavailable to accommodate refugees for any reason [44].

These studies show a consensus to strengthen the use of urban resources and ensure the safety of emergency shelters as much as possible. In any case, there are several types of hazards in cities, which include natural disasters, accidents, public health events, and lack of social security. It is crucial that preparedness for multi-hazards is strengthened in order to save lives, reduce injuries, and limit property damage [45]. In 1999, Federal Emergency Management Agency (FEMA) established the Guide for All-Hazard Emergency Operations Planning, which highlights preparedness, response, and short-term recovery planning elements. The guide encourages managers to address all of the hazards in a single EOP instead of relying on stand-alone plans. It also helps emergency management organizations produce EOPs that serve as the basis for effective response to any hazard and facilitates integration of mitigation into response and recovery activities. The key issues in the process include profiling hazards and their potential consequences, figuring out what kinds of resources and facilities can be brought to emergency response and recovery, and assess geographic areas prone to be affected by hazards, vulnerable facilities, and distribution of populations exposed to hazards [46]. Besides planning, managing land use to accomplish hazards mitigation is also a practical method in which the hazards can be reduced to a scale that can be borne by governments, communities, and individuals [47]. In general, studies based on multi-hazard environments are less than those that responded to a certain type of disaster. Shelter locations are likely to be unsuitable or insecure for refugees when facing multiple disasters. For example, in a scenario of simultaneous earthquake and flood, some of the earthquake shelters may be flooded.

Using the GIS platform, this study explores the selection and allocation of urban resources for emergency shelters in the context of a multi-hazard environment. The manuscript is organized into four sections. The first presents an overview of the study area, as well as a description of the main hazards along with the characteristics and details of methods, including physical suitability assessment of overall urban resources, spatial analysis of the affected area, and prediction of the affected population under various disaster scenarios. Results and findings follow in the next section. There are some deficiencies and limitations in the assessment, prediction, and allocation, which are discussed in the third section. Conclusions and future study are presented at the end. We hope that this work provides a reference for the construction of emergency shelters and helps form EOPs to mitigate the impact of hazards.

## 2. Materials and Methods

### 2.1. Overview for the Study Area

**Figure 1:**
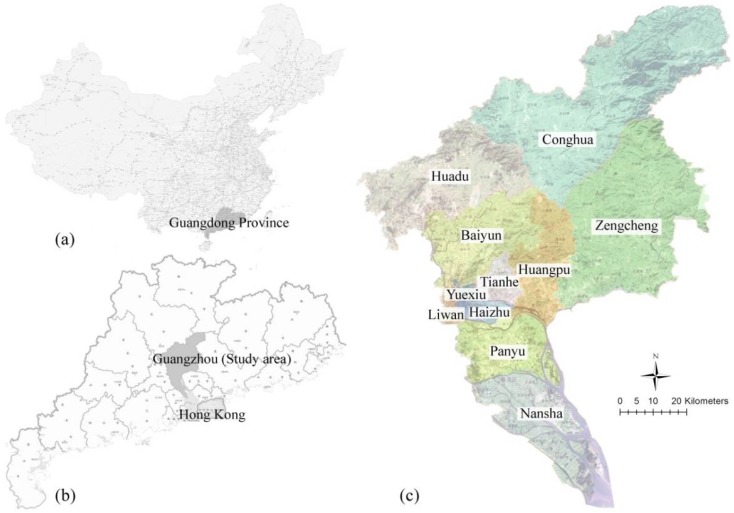
Location of (**a**) Guangdong Province in China; (**b**) Guangzhou (study area) and (**c**) 11 districts in Guangzhou.

Guangzhou, the capital of Guangdong Province, is located on the northern edge of the Pearl River Delta, facing the South China Sea and close to Hong Kong (see Figure 1). It has an area of 7434.4 km^2^ in 11 districts and a residential population of 14.5 million at the end of 2017. According to official statistics, as one of the most disaster-prone cities in China, it is struck regularly by typhoons, storm surges, floods, lightning, geological disaster, and other hazards, which has caused heavy life and economic losses (see Table 1). In addition, due to high population density, economic factors, and a large number of buildings, Guangzhou is becoming more vulnerable to complex disaster environments than smaller cities. Authorities have made efforts in disaster reduction and have built emergency shelters for earthquake. Although the city has several resources that can be used as shelters; uneven distribution, insufficient quantitative analysis, and gaps from national standards are practical issues that need to be further studied. From a disaster history perspective, typhoons hit Guangzhou at high frequency and are usually accompanied by storm surges and floods, which is why it causes serious damages. Lightning disasters and fires do not require specialized emergency shelters or, if necessary, nearby facilities can be used as replacements. According to records, the strongest earthquake in Guangzhou occurred in 1940 and its surface wave magnitude (Ms) was 5.0 while the most recent earthquake was in 2015 with a magnitude of 1.9. Geologically, Guangzhou is not located in a high-risk earthquake zone; however it is important to be prepared due to the unpredictable nature of earthquakes. In addition, other hazardous locations of geological disaster, dangerous chemicals, and explosives also need to be excluded to ensure the safety of emergency shelters.

### 2.2. Methods

#### 2.2.1. Assessment of Urban Resources for Emergency Shelters

Parks, squares, schools, stadiums, and other public facilities are potential resources for emergency shelters, but not every one of these is physiologically or socially suitable [18]. Based on a survey of Guangzhou, candidate shelters mainly consist of green spaces, city squares, school playgrounds, gymnasiums, educational institutions, and community centers (see Table 2). These facilities were geocoded by GIS to produce a geographic shelter database. However, new shelters need to be added to the list because the available resources are unevenly distributed across the city, and affected persons may not be able to access them on time. Therefore, it is necessary to carry out an overall assessment of urban resources, excluding unsuitable candidate shelters and hazardous areas as much as possible and selecting available and reliable resources for emergency shelters. Its immediate effect is to reduce losses both in primary and secondary disasters.

The selection of factors for this assessment primarily considers primary disasters, as well as some serious secondary ones. Earthquakes, floods, and storm surges should be given priority in consideration of the disaster history of Guangzhou. The factors vary with different disasters; however, the common factors are geological environment, gas stations, natural gas fueling stations, high voltage lines, power plants, dangerous goods warehouse, high pressure gas pipelines, terrain slope, waters, heritage conservation areas, and waste treatment stations [17]. Tsunami inundation areas should be taken into account during earthquakes, because they could follow. For flood and storm surges, flood levels and terrain elevation need to be considered to find areas that are easily submerged. Table 3 shows the factors and standards of assessment [48]. Unsuitable means locations that cannot be used as shelters; neutral stands for spare locations; and suitable represents locations that can be used.

Specific calculations will be implemented in the GIS platform. According to the factors in Table 3, single-factor evaluation maps can be produced with buffer analysis. The assessment result of each type of disaster is then generated by using the minimum value superposition method, which is modeled from Liebig’s law (expressed in Equation (1)) [49]. Final results help to select existing resources and new available areas, which are suitable for emergency shelter locations:

(1) $$S=Min\left(x_{1},\;x_{2},\;\ldots \;,\;x_{n}\;\right)$$

where *S* is the final assessment score, *x_n_* (*n* = 1, 2, …, *n*) is the single factor score calculated by buffer analysis, according to the criteria in Table 3, and each factor plays “bottom line” role. “*Min*” determines safe locations for shelters by overlay analysis of a single factor.

#### 2.2.2. Shelter Demand Prediction

Shelter demand can be predicted by the spatial distribution of affected people, which also provides a direct basis for allocation of emergency shelters. For the sake of simplicity, three assumptions are presented below [30].

(1) The demand only considers people directly affected by the disasters. Those who are not in the affected areas are not counted as potential evacuee.
(2) The population of a community is evenly distributed. The ideal situation is to get everyone’s exact location to accurately allocate the limited resources.
(3) Everyone directly affected by a disaster will take refuge in a nearby shelter and can only stay in one shelter, reducing the deviation between shelter supply and demand as much as possible. Complex evacuation behaviors are not considered [27].

**Figure 2:**
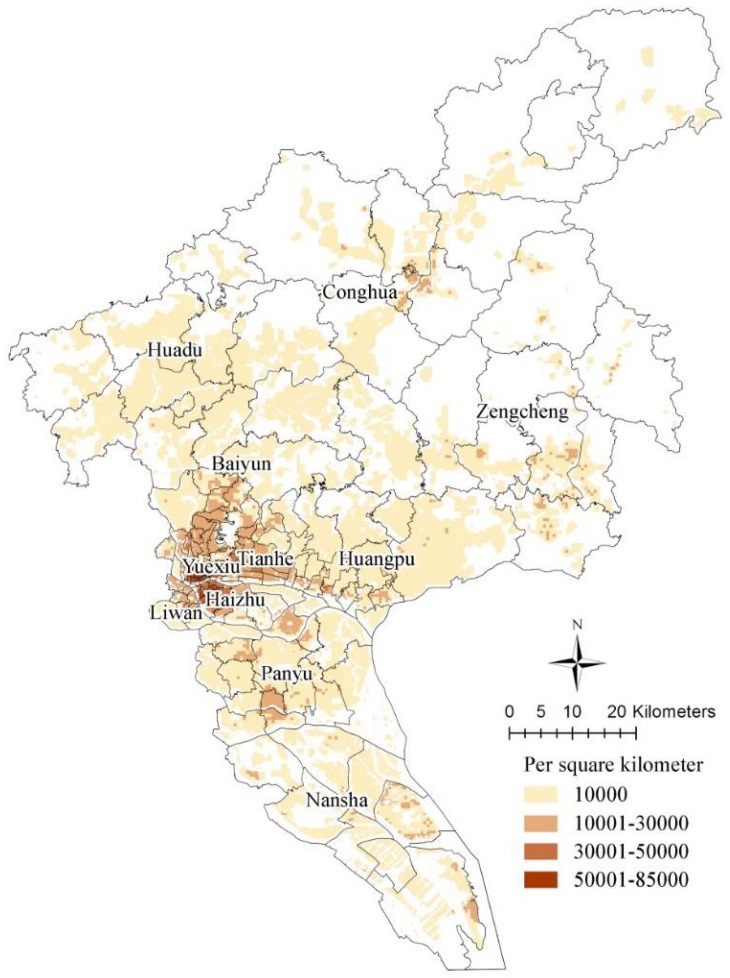
Planning community population density by 2020 (Source: Guangzhou City Master Plan for 2011–2020).

The basic principle of demand prediction is to calculate the population in the affected areas and complete the entire analysis on the GIS platform. In order to ensure foresight and consistency with local urban planning, population distribution is based on data from the Guangzhou City Master Plan for 2011–2020 (see Figure 2).

For floods and storm surges, areas less than 3 m above sea level and areas covered by 3 m of tide are identified as submerged area, according to international experience and disaster history in Guangzhou. Geographic data of the submerged areas on the basis of the digital elevation model (DEM) of Guangzhou should first be produced. Demand can then be calculated by overlay analysis with spatial distribution of population density.

The demand for more shelters during earthquakes is huge. Collapsed houses force large numbers of homeless people to seek refuge. Based on China’s earthquake prevention practices and the construction standards of existing buildings in the early years, 85% of existing buildings will stay intact and 15% will be moderately damaged when a standard fortified earthquake (Ms 7.0) occurs. About 80–90% will be moderately or severely damaged and only 10% will stay intact when it is a huge one (Ms 9.0) [50]. The number of affected people in an earthquake can normally be calculated by Equation (2). First, the ratio of affected people to the total population is calculated with the proportion of damaged buildings that have been set before. Then, according to the spatial distribution of the population in the city (see Figure 2), the affected population in each district can be estimated:

(2) $$M=1/a\times \left(2/3\times A_{1}+A_{2}+\times 7/10\times A_{3}\right)$$

where *M* is the number of affected people, *a* is per capita residential area (m^2^), *A*_1_, *A*_2_, and *A*_3_ represent the area of collapsed, severely damaged, and moderately damaged residential buildings (m^2^), respectively.

## 3. Results and Findings

**Figure 3:**
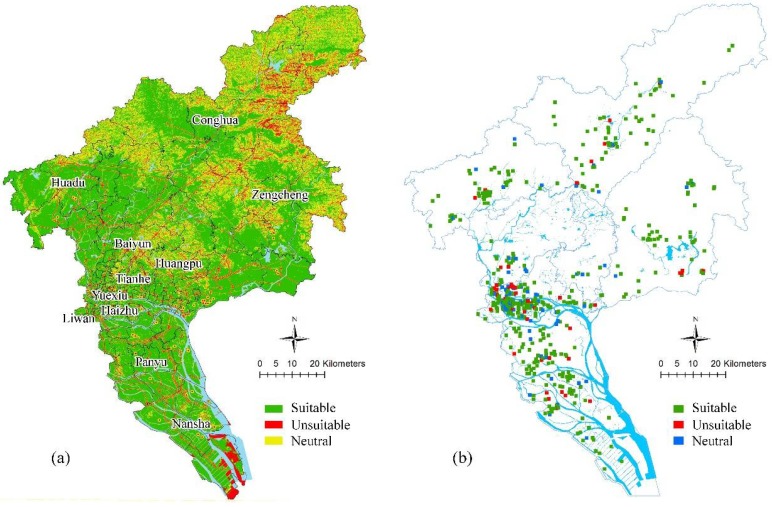
Assessment of (**a**) overall urban resource and (**b**) facilities considered as candidate shelters in a multi-hazard environment.

Suitable shelters that respond during floods, storm surges, and earthquakes are located in most areas of central districts, including Yuexiu, Liwan, Tianhe, Haizhu, Huangpu, and Panyu. However, attention should be paid to factors such as high voltage lines, refueling stations, and more. The eastern part of Baiyun, the northern part of Huadu, Northeastern Conghua, and Northwestern Zengcheng are not suitable for the construction of emergency shelters due to topography and geological disasters. The southern part of Nansha is likely to be submerged in a storm surge because of its low altitude. Considering secondary disasters (tsunami after earthquake), shelters should not be located here too. When a flood level is more than 2.5 m in the eastern part of Nansha, there is a certain probability that it will be submerged, deeming it inappropriate for flood-based shelters; similar is the case with the low-lying areas along the Pearl River in the city (see Figure 3a).

According to the superposition analysis of overall resource assessment and existing shelter geographic databases consisting of current available facilities, the points suitable for shelters and the spare ones can be selected (see Figure 3b). In general, the spatial distribution of existing available resources is uneven and differs a lot between different districts.

The number of outdoor shelters in Panyu is 344, which is the highest and accounts for 17.8% of the total. This is followed by Zengcheng, which accounts for 15.0%. Liwan has the least number of 43, which accounts for only 2.2%. From the area point of view, Panyu has the largest area of available resources, which is about 1413 hectares and accounts for 25.1% of the total. This is followed by Haizhu, which accounts for 12.4%. Nansha has a small area of only 137.5 hectares, which accounts for 2.5%. Panyu has the largest number of indoor candidate shelters — 101 available resources, which accounts for 17.1% of the total. This is followed by Tianhe and Conghua, which account for 12.0% and 11.9%, respectively. The number of resources that can be used as emergency shelters in Liwan is the least with 17%, and only 2.9% of the total. In terms of area, Panyu has the largest area of available resources, which is about 541.6 hectares and accounts for 21.1% of the total. This is followed by Tianhe, which accounts for 21.0%. Liwan has the smallest area of only 23.5 hectares, which accounts for 0.9% (see Table 4).

**Figure 4:**
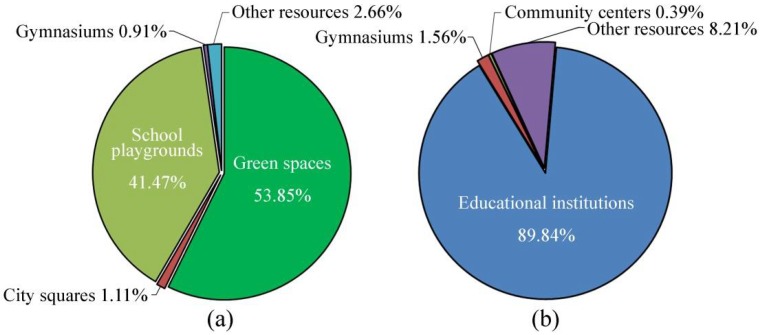
Composition of (**a**) outdoor available resource (**b**) indoor available resource.

With regard to the type of outdoor resources, the proportion of green spaces is the largest and accounts for 53.85% of the total, followed by school playgrounds accounting for 41.47%. City squares and gymnasiums have a small proportion of 1.11% and 0.91%, respectively (see Figure 4a). Green spaces are the most frequently used spaces by citizens and can accommodate disaster relief facilities as well as effectively block fires and other secondary disasters. Therefore, green spaces should be given priority as refuge and be considered as an outdoor emergency shelter. School playgrounds are outside the collapsed area of the building and all can be used for emergency shelter. Most existing schools are evenly distributed with a service radius between 500 m and 1000 m, which makes them easy to reach within a short time frame. For indoor available resources, educational institutions occupy an absolute majority of the total with a proportion of 89.84%, owing to the high number of universities, colleges, and schools in Guangzhou (see Figure 4b). Meanwhile, indoor areas of gymnasiums, hospitals, welfare facilities, and public security facilities should not be ignored, since these facilities can provide support in disaster relief or post-disaster reconstruction.

**Figure 5:**
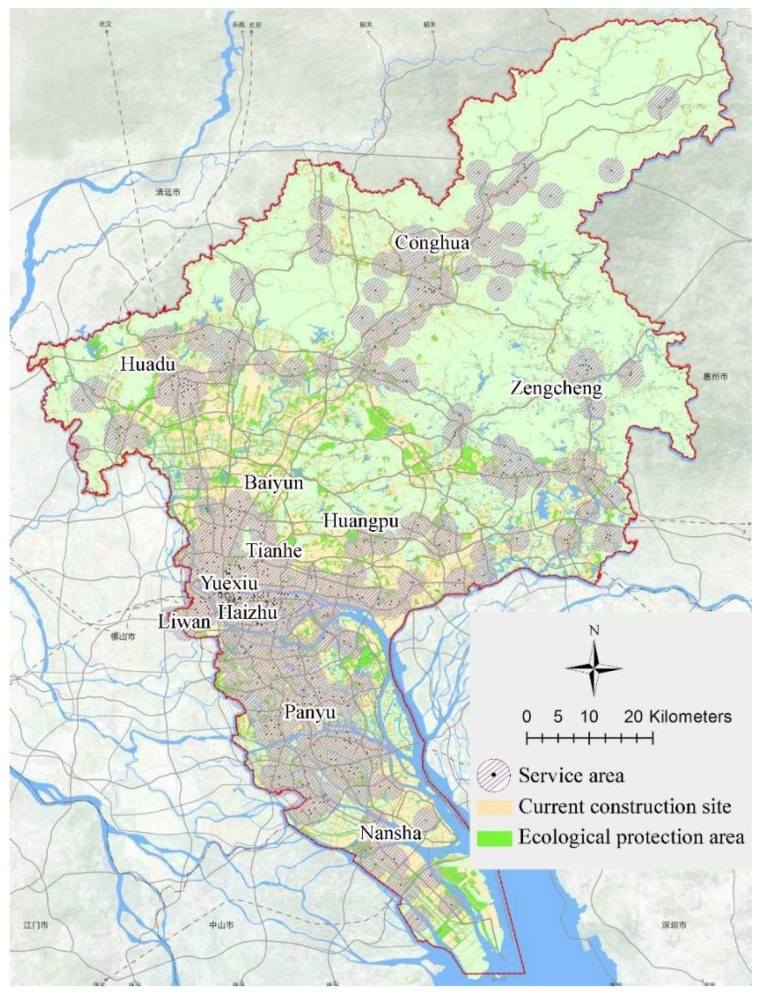
Service area of the candidate shelters selected from existing available resources.

The area of existing resources that can be used as emergency shelters is 9230 hectares and per capita effective sheltering area has reached 7.19 m^2^, which is far more than the minimum requirement in the national standard of 1.5–2 m^2^ per person. This means that the total amount of existing available resources can meet residents’ demand for emergency shelters. However, there is a major problem that needs to be pointed out. These resources are unevenly distributed and does not cover all urban built-up areas, which could result in residents who are not covered being unable to reach shelters on time, causing significant losses. There exists a lot of blind areas for emergency shelter services. Figure 5 shows the candidate shelters’ service area, which is described as a service radius of 2000 m.

**Figure 6:**
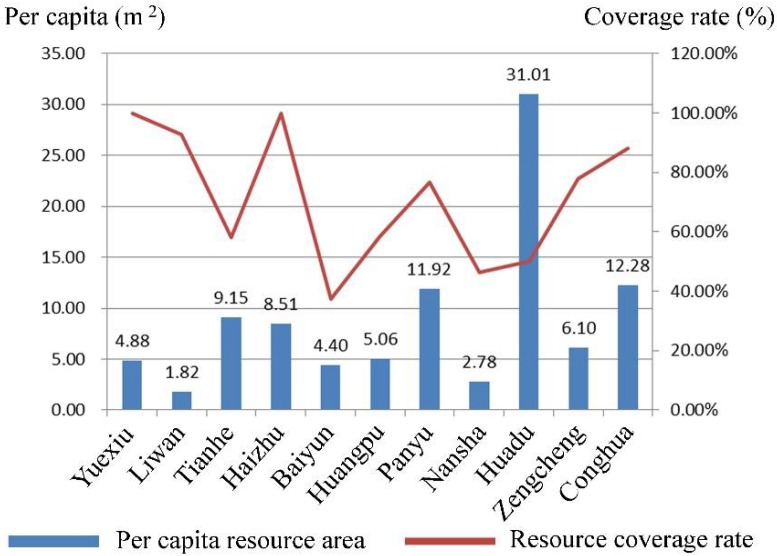
Existing per capita available resource area and coverage rate.

Figure 6 shows the existing per capita available resource area and coverage rate of all 11 districts in Guangzhou. Overall, only Yuexiu, Liwan, Haizhu, and Conghua have a coverage rate of more than 80%; it is lesser than 50% in Baiyun and Nansha. In Yuexiu and Liwan, despite a considerable resource coverage rate, the per capita area is less than 5 m^2^ and Liwan presents a state of resource shortage with an average area of 1.82 m^2^, lower than the national minimum standard due to high population density, which can cause supply shortages of emergency shelters. Such areas must tap into more available resources in order to meet national standard requirements. The per capita area of Tianhe, Panyu, Zengcheng, and Huadu is sufficient, but due to the uneven distribution of available resources, the coverage rate is low—58.09%, 76.79%, and 50.08%, respectively. These districts need to add a certain number of emergency shelters in order to increase the coverage of population and urban construction activities. Due to the fact that Baiyun, Huangpu, and Nansha have more hazards and lag behind other districts in overall development, their per capita area and coverage rate is weak. One of the common features of these districts is that the land reserves are more abundant than others, enabling authorities in urban planning to increase the number of facilities that can be used as shelters.

**Figure 7:**
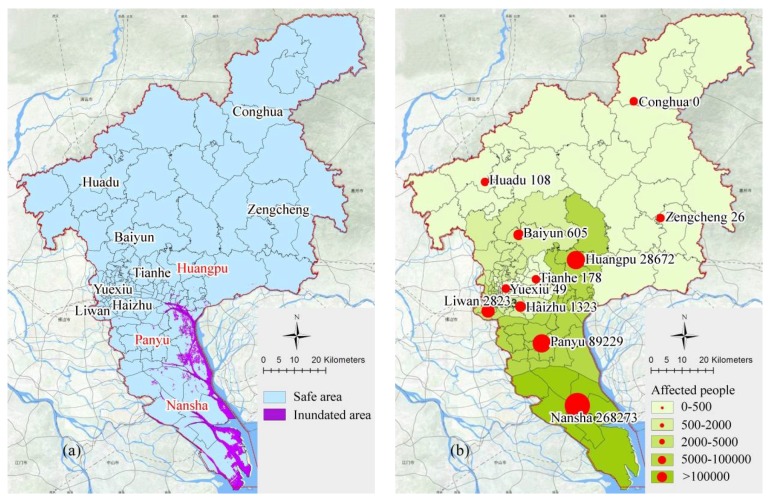
Distribution of (**a**) inundated area and (**b**) affected people during floods.

Figure 7 shows areas inundated during floods; they are mainly distributed in the southern part of Nansha with a lower elevation when flood water level is 3 m, which has the largest number of affected people. The number of affected people is also high in areas near the sea inlet of Panyu and the low-lying areas of Huangpu because of high population density. The selection of shelters in these areas can be slightly biased towards indoor shelters and more consideration should be given to areas with higher ground. The northern part of Guangzhou includes Conghua, Zengcheng, and Huadu, which are almost unaffected by flood due to their high altitude. However, this does not mean that it has no need for emergency shelters. Taking into account the sudden nature of heavy rains, flood shelters in these areas can be set up together with earthquake-based shelters. During a storm surge, the inundated area and affected people are highest in Nansha, followed by Panyu and Huangpu. Since storm surges mainly hit southern Guangzhou, meteorological disasters are more complicated and secondary disasters are also likely to occur. The selection of emergency shelters in these areas requires consideration of the resilience of meteorological disasters in addition to preference for higher-lying indoor locations. According to earthquake analyses, the number of affected people in each district will increase as the permanent population increases. It should be pointed out that old urban areas including Yuexiu, Liwan, and Haizhu will have a high instantaneous demand for emergency shelters and evacuation will be very difficult because of the high population density and the large number of old houses. The scale of the inundated area and the number of affected people in floods, storm surges, and earthquakes is presented in Table 5.

Table 5 also shows that the number of affected people in both standard earthquakes and high-intensity ones is far more than in floods and storm surges. Considering the shortage of urban resources and the low probability of major earthquakes in Guangzhou, the prediction of earthquake shelter demand in this study is based on the condition of standard intensity. When a major earthquake strikes, people can use outdoor spaces for temporary refuge as a supplement to emergency shelters. At the same time, it also prompts an increase in the number of facilities that can be used as shelters when carrying out urban renewal projects. The shelter demand prediction for floods, storm surges, and earthquakes is shown in Table 6 in combination with the distribution of affected people and per capita area requirement as per national standards.

**Figure 8:**
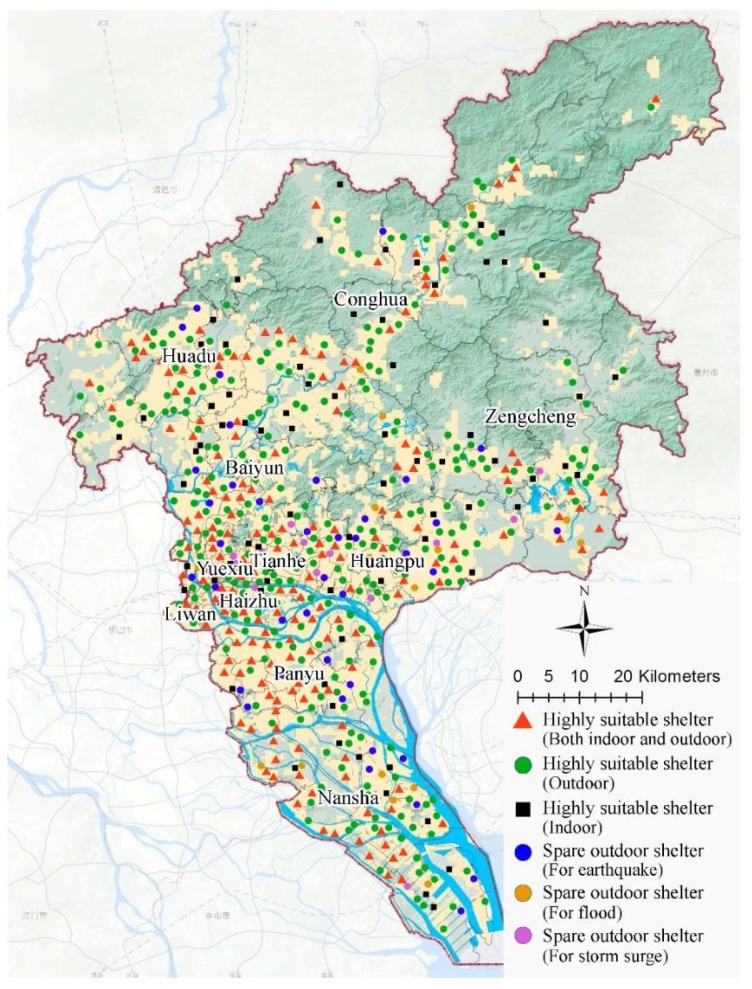
Allocation of emergency shelters in a multi-hazard environment.

According to the results of the previous analysis, the spatial allocation of emergency shelters in response to floods, storm surges, and earthquakes can be achieved on the GIS platform. In order to ensure that a large majority of the affected people are covered, a service radius of 1000–2000 m is set for preliminary analysis. Furthermore, combined with the overall assessment of urban resources done before, the suitable (safe) locations can be used for the allocation of highly suitable shelters, which means that neither indoor shelters nor outdoor shelters here will be destroyed or threatened by other secondary disasters in most scenarios, even if an earthquake (not a huge one), a storm surge, and a flood hit at the same time. In order to be flexible, spare ones are also marked out for use in special situations. They can also withstand two kinds of disasters under normal circumstances. Considering that it may cause some confusion in practice, spare shelters are set to respond to earthquakes, storm surges, and floods separately, depending on the area where they are located. Figure 8 shows the distribution of highly suitable shelters and spare shelters in a multi-hazard environment.

## 4. Discussion

The strain of urban resources and the increasing complexity of the disaster environment are the origins of this study. The reality of limited urban resources and unpredictable losses in disasters does not allow us to make obvious mistakes in hazard-prevention activities. This work offers two contributions that are helpful in forming EOPs. The first and most important one is ensuring that the shelters avoid destruction (or are not seriously threatened)—by selecting and assessing urban resources based on a multi-hazard environment. The other one is an estimation of potential damage (affected areas and populations), which can help identify locations where the demand for shelters is large. This also provides a reference for management departments to allocate resources in pre-disaster preparedness and disaster relief operations. However, there are deficiencies and limitations.

Physical and social suitability of candidate shelters should be included in the assessment of available resources. Our work on social suitability is not enough. The former is mainly to ensure the safety of land use with the main considerations to avoid hazard sources and secondary disasters areas. The latter mainly considers the proximity to demand centers and public facilities and whether they can get support quickly [18]. One of the effects of the social suitability assessment is the certain influence on the ranking of selected resources that can be used as shelters. For example, shelter A and B are screened out by using the physical suitability assessment and it is assumed that A is closer to a hospital. If social suitability is not considered, there is no difference between them. If considered, then A is more likely to be given priority. Another effect is that the number of available resources might not be so large because some areas are not close to the support center and may be identified as inappropriate resources. Considering the shortage of resources in Guangzhou, the social suitability assessment may cause a problem of insufficient emergency shelters, which means that the factors in this assessment need to be adjusted, according to different cities [17]. Additionally, it can be optimized when making EOPs [46].

There are two limitations that need to be addressed in shelter demand prediction. One is caused by the nature of the city’s population. Shelter demand should consider the distribution of population during the day and night [30,31]. The approach the authors took to predict the affected population by using the distribution of resident population tends to be suitable for disasters that occur at night. In other words, the lack of consideration for the daytime population will result in a certain bias in the results. In big cities, such as Guangzhou, the large commuting population indicates that emergency shelters in some areas may be insufficient to respond to daytime disasters. Therefore, a final and ideal prediction should be based on the maximum peak in the day and at night, but it is difficult to obtain data for daytime population distribution. Another limitation is that the damage assessment in earthquakes was calculated by an approximate method, which is macroscopic and simplified. Under normal circumstances, earthquake loss estimation needs a more realistic simulation environment including data of geology, buildings in each block and the population, roads, potential sources of danger, natural environment, etc. [33]. In Guangzhou and even China as a whole, the geodatabase for the above-mentioned information is lacking, making it almost impossible to estimate the details of earthquake losses.

In the allocation of emergency shelters, due to the large spatial scale of the study area and the lack of road network information, the authors assessed the coverage of shelters for affected people with a service radius, rather than real distance or evacuation routes, which puts forth two problems: First, it underestimates the time required for people to reach a shelter and overestimating the coverage of evacuation sites. A suitable solution is to make an accessibility assessment that is often used in small-scale and medium-scale areas. Accessibility analysis refers to the difficulty of entering an emergency shelter through evacuation routes [23]. Additionally, an effective premise of the accessibility assessment is that the evacuation routes are known. Therefore, it is necessary to assess the capacity of urban roads during disasters and identify the roads that can be used as evacuation routes, usually implemented in the event of a destructive disaster [42,43,44]. However, at the level of the entire city of Guangzhou, the use of the service radius to describe the coverage of shelters offers strong guidance in solving the problem of insufficiency and allocation of emergency shelters. A study on the accessibility should be conducted at relatively small spatial scales, such as district-level and community-level scales.

## 5. Conclusions

With rapid expansion of Chinese cities over the years and the high concentration of population in urban areas, the entire society is becoming vulnerable to disasters, making it absolutely necessary to build a disaster prevention system represented by the construction of emergency shelters. Unlike current studies that focus on responding to earthquakes or other types of disasters, this paper explored the methods of urban resource selection, estimation of the affected area and population, and shelter demand prediction in GIS for a multi-hazard environment. The analysis of the Guangzhou case provides an enlightening reference for future study, planning and construction of emergency shelters in other cities in response to various disasters, and the formation of EOPs.

Through an overall suitability assessment of urban resources for floods, storm surges, and earthquakes, the areas suitable (or unsuitable) for shelter locations can be identified. The results show that, although there are sufficient available resources for emergency shelters in Guangzhou, the distribution of resources, the per capita effective area, and the resource coverage rate are quite different among the 11 districts, which will result in some of the affected people being unable to find a shelter nearby to take refuge in during a severe disaster. In view of the serious problem in the downtown area, including Yuexiu, Haizhu, Liwan, and Tianhe, such areas with high population density should tap into more stock resources in future urban renewal. Other districts can add new facilities that can be used as emergency shelters in future urban constructions, such as schools, parks, and gymnasiums. The prediction of disaster-affected areas tells us the approximate spatial distribution and quantity of the affected people so that different tactics can be used to allocate shelter resources in each district. The analysis shows that areas with a high population density and a high number of old buildings have a large demand for shelters due to earthquakes and a high demand in areas with low altitudes, close to estuaries, and the low-lying region along the Pearl River when facing floods and storm surges. Lastly, according to the results of resource assessment and demand prediction, the allocation of emergency shelters was done for the entire city, in order to cover the affected population. In addition, spare shelters are selected and marked in order to be flexible and withstand possible extreme disasters. However, unavoidable issues discussed above (social suitability in resource assessment, population distribution during typical time periods, and accessibility of shelters) will have an impact on the results of the study, and need to be further studied in medium-scale or small-scale areas.

## Figures and Tables

**Table 1:** Main hazards in the study area and their characteristics.

Hazards	Intensity	Affected People	Damages
Typhoon “Rainbow” in 2015	A maximum wind force near the center at landing of 50 m per second	3 dead and 170 thousand people transferred in the province	3374 houses collapsed and a direct economic loss of 23.24 billion CNY in the province
Storm surge caused by typhoon “Hagupit” in 2008	A highest tide level of 2.72 m	20,478 people transferred	99 villages flooded and 102 km^2^ of farmland affected
Floods caused by catastrophic rain in 2014	A maximum 1 hour rainfall of 93.9 mm	7 dead, 1 missing, and 42,500 people transferred	6810 houses collapsed and a direct economic loss of more than 700 million CNY
181,000 lightning in the whole year of 2010	25 lightning per square kilometer	4 dead and 2 injured	110 accidents and an economic loss of 5.89 million CNY
2644 fires in the whole year of 2015	No major fire	22 dead and 12 injured	A direct economic loss of 28 million CNY
1270 geological disasters in the whole year of 2008		1 dead and 1308 people transferred	20 houses collapsed and a direct economic loss of more than 15.3 million CNY
Destructive earthquake in “Nanhai county” in 1940	Ms 5.0	No statistics	No statistics
Dangerous chemicals and explosives		Potential hazards	Potential hazards

Source: Official post-disaster statistics.

**Table 2:** Resource types considered as candidate shelters.

Facility as Outdoor Candidate Shelter		Facility as Indoor Candidate Shelter	
Facility	Types of Facility	Facility	Types of Facility
Green space	Park, street green area, protection greenbelt	Educational institution	University, college, middle school, primary school
City square	Public square, memorial plaza, other large squares	Gymnasium	Indoor venue of gymnasium
School playground	Playground of university, college, middle school, and primary school	Community center	Indoor area of community Center
Gymnasium	Outdoor venue of gymnasium	Other facilities	Indoor area of hospital, welfare facility, and public security facility
Other facilities	Outdoor area of library, community center, medical center, welfare facility, and others		

**Table 3:** Factors in the assessment of urban resources for emergency shelters.

Factors	Criteria	Comment
Sea level height in tsunami, tide level in storm surge, flood level	>2.5 m	Unsuitable
	2.5–1.5 m	Neutral
	<1.5 m	Suitable
Geological environment	Geologically inappropriate areas and near-fault region	Unsuitable
	Others	Suitable
Distance from gas stations, natural gas fueling stations, high voltage lines, and power plants	<50 m	Unsuitable
	50–100 m	Neutral
	>100 m	Suitable
Distance from dangerous goods warehouse	<500 m	Unsuitable
	500–1000 m	Neutral
	>1000 m	Suitable
Distance from high pressure gas pipelines	<25 m	Unsuitable
	25–50 m	Neutral
	>50 m	Suitable
Terrain slope	>30°	Unsuitable
	15–30°	Neutral
	>15°	Suitable
Distance from waters	0–30 m	Neutral
	>30 m	Suitable
Terrain elevation	<1.5 m	Neutral
	>1.5 m	Suitable
Distance from heritage conservation areas	0–30 m	Neutral
	>30 m	Suitable
Distance from waste treatment stations	<100 m	Neutral
	>100 m	Suitable

**Table 4:** Existing available resources as a candidate shelter.

District	Outdoor Candidate Shelter			Indoor Candidate Shelter		
	Number (a)	Land Area (ha) (b)	Percentage (a), (b)	Number (c)	Building Area (ha) (d)	Percentage (c), (d)
Yuexiu	51	165.8	2.6%, 3.0%	26	56.7	4.4%, 2.2%
Liwan	43	171.1	2.2%, 3.0%	17	23.5	2.9%, 0.9%
Tianhe	121	544.1	6.3%, 9.7%	71	539.8	12.0%, 21.0%
Haizhu	94	695.8	4.9%, 12.4%	62	426.0	10.5%, 16.6%
Baiyun	274	663.1	14.2%, 11.8%	44	271.1	7.5%, 10.5%
Huangpu	70	457.4	3.6%, 8.1%	33	27.2	5.6%, 1.1%
Panyu	344	1413.1	17.8%, 25.1%	101	541.6	17.1%, 21.1%
Nansha	128	137.5	6.6%, 2.5%	76	56.1	12.9%, 2.2%
Huadu	244	466.0	12.6%, 8.3%	42	239.4	7.1%, 9.3%
Zengcheng	290	415.3	15.0%, 7.4%	48	182.5	8.1%, 7.1%
Conghua	277	493.5	14.3%, 8.8%	70	204.1	11.9%, 8.0%

**Table 5:** Scale of inundated area and affected people during floods, storm surges, and earthquakes.

District	Flood		Storm Surge		Earthquake	
	Inundated Area (ha)	Affected People	Inundated Area (ha)	Affected People	Affected People by Standard Intensity	Affected People by High Intensity
Yuexiu	—	—	—	—	100,000	520,000
Haizhu	49.29	1323	—	—	200,000	930,000
Liwan	80.12	2823	—	—	150,000	560,000
Tianhe	3.20	178	—	—	150,000	830,000
Baiyun	4.37	605	—	—	240,000	1,480,000
Huangpu	1572.04	28,672	1258.48	9629	160,000	1,060,000
Huadu	3.08	108	—	—	190,000	1,030,000
Panyu	6984.22	89,229	6452.87	76,075	190,000	1,030,000
Nansha	14,186.22	268,273	16,836.51	394,514	160,000	1,100,000
Conghua	—	—	—	—	100,000	540,000
Zengcheng	—	—	—	—	250,000	1,250,000
Total	22,882.54	391,211	24,547.86	480,218	1,890,000	10,330,000

**Table 6:** Shelter demand prediction in multi-hazard environment.

District	Flood Shelter (ha)	Storm Surge Shelter (ha)	Earthquake Shelter (ha)
Yuexiu	—	—	40
Haizhu	0.53	—	80
Liwan	1.13	—	60
Tianhe	0.07	—	60
Baiyun	0.24	—	96
Huangpu	11.47	3.85	64
Huadu	0.04	—	76
Panyu	35.69	30.43	76
Nansha	107.31	157.81	64
Conghua	—	—	40
Zengcheng	—	—	100
Total	156.48	192.09	756
